# Latencies to the first interictal epileptiform discharges recorded by the electroencephalography in different epileptic patients

**DOI:** 10.1186/s12883-023-03474-2

**Published:** 2023-12-01

**Authors:** Chenyu Liu, Yi Qi, Liang Wang, Ce Zhang, Li Kang, Suhang Shang, Jingxia Dang

**Affiliations:** Department of Neurology, The First Affiliated Hospital of Xi’an Jiaotong University, 277 West Yanta Road, Xi’an, Shaanxi, 710061 China

**Keywords:** Epilepsy, Electroencephalography, Interictal epileptiform discharge

## Abstract

**Purpose:**

Interictal epileptiform discharges (IEDs) captured in electroencephalography (EEG) have a high diagnostic value for epileptic patients. Extending the recording time may increase the possibility of obtaining IEDs. The purpose of our research was to determine how long it took for various epileptic individuals to receive their first IEDs.

**Methods:**

We retrospectively analyzed patients who were diagnosed with epilepsy and had no anti-seizure medications (ASMs) between September 2018 and March 2019 in the neurology department of the First Affiliated Hospital of Xi'an Jiaotong University. Each individual underwent a 24-h long-term video electroencephalographic monitoring (VEM) procedure. Clinical information including age, gender, age of seizure onset, frequency of seizures, the interval between last seizure and VEM, and results of neuroimaging were gathered. We also calculated the times from the start of the VEM to the first definite IEDs.

**Results:**

A total of 241 patients were examined, including 191 with focal-onset epilepsy and 50 with generalized epilepsy. In individuals with focal-onset epilepsy, the median latency to the first IED was 63.0 min (IQR 19.0–299.0 min), as compared to 30.0 min (IQR 12.5–62.0 min) in patients with generalized epilepsy (*p* < 0.001). The latency to the first IED is significantly related to the age of seizure onset (HR = 0.988, *p* = 0.049), the interval between last seizure and VEM (HR = 0.998, *p* = 0.013). But it is not correlated with seizure frequency, gender and age.

**Conclusions:**

IEDs were discovered during 24-h EEG monitoring in 222/241(92.1%) of the epilepsy patients that were included. Compared to focal-onset epilepsy, generalized epilepsy demonstrated a much shorter latency to IED. Patients with late-onset epilepsy or those without recent episodes may require longer EEG monitoring periods.

## Introduction

Electroencephalography is a critical test for epilepsy diagnosis, classification, and presurgical evaluation. It is also an important factor in deciding when to start anti-seizure medication (ASM) treatment or when to withdraw drugs. Commonly used EEG techniques include routine EEG and long-term video EEG. Routine EEG sessions typically last 40 min while the patient is awake. The duration of long-term video-EEG ranges from several hours to several days, depending on the patient's needs. And, for almost all patients with paroxysmal symptoms suspected or already diagnosed with epilepsy, we perform the VEM, which lasts 24 h and includes experiments such as eye opening, eye closing, hyperventilation, and intermittent photic stimulation.

Interictal epileptiform discharge captured in EEG is critical evidence for the diagnosis of epilepsy in patients suspected of having it. The short-term EEG test may miss IEDs that occur after the examination is completed. According to a previous study, the positive rate of routine EEG is only 12.1% [1]. By extending the monitoring time, we can increase the positive rate. The monitoring time of EEG cannot be extended indefinitely due to patient tolerance and economic burden, as well as the rational use of medical resources. We can capture IEDs soon after EEGs begin in some patients, but some IEDs come late after several hours or even longer. In epileptic patients, the latency to the initial epileptic discharge can range from several minutes to several days. We sought to find the relationship between clinical data and the latency to the initial epileptic discharge during the video EEG examination in epileptic patients.

## Methods

### Patient recruitment

This is a retrospective study that included 24-h VEM recordings of epileptic patients performed between September 2018 and March 2019 in the neurology department of The First Affiliate Hospital of Xi’an Jiaotong University. We include patients aged 6 to 90 who did not have a prior diagnosis of epilepsy and have not taken any ASMs in the previous three months. We excluded patients who had a definite seizure within the previous 24 h, as well as those who had acute symptomatic seizures caused by stroke or a central nervous system infection.

We collected the following data: age, gender, age of seizure onset, seizure frequency, interval between last seizure and VEM, and neuroimaging results.

All patients were examined using NIHON KOHDEN EEG-1200C 28-channel surface video-EEG systems for 24 h. The electrodes were placed in accordance with the international 10–20 system [2]. The 28 electrodes include Fp1/2 F3/4 C3/4 P3/4 O1/2 F7/8 T3/4 T5/6 Fz Cz Pz, two electrodes for eye movement, two electrodes for EMG, two electrodes for ECG, two reference electrodes placed at two earlobes, and the grounding electrode placed at Fpz. EEG was sampled at 500 Hz, filtered at 0.1–70 Hz, had an impedance of less than 5kΩ, and was typically reviewed at 10 mV/mm, 30 mm/s. Prior to our VEM, our patients had a natural awake-sleep cycle with no sleep deprivation. The majority of patients begin the VEM examination between 8 and 12 a.m. We conducted the evoked experiments, which included eye opening and closing, hyperventilation, and photic stimulation, immediately after obtaining stable EEG signals. Following the completion of the VEM, the EEG was visually analyzed by two experienced neurologists. The International Federation of Clinical Neurophysiology (IFCN) [3] criteria were used to identify IEDs. The latency of the first definite IED was calculated. Two experienced neurologists determined whether the patient had epilepsy and, if so, what type of epilepsy he or she had. The International League Against Epilepsy (ILAE) 2017 classification [4] was used to classify epilepsy. Patients who could not be classified as having focal or generalized epilepsy were not evaluated.

The Statistical Package for the Social Sciences (SPSS) for Windows, version 23.0, was used for statistical analyses (IBM Corporation, Armonk, NY, USA). The normally distribution of the variables was determined using analytical methods (Kolmogorov–Smirnov/Shapiro-test). For non-normally distributed data, descriptive analyses were presented using the median and IQR. For nonparametric data, the Mann–Whitney U-test was used, and for ratios, the chi-squared test was used. The Kaplan–Meier, Log Rank test, and COX multivariate analysis were used to assess the relationship between IED latency and gender, age, age of onset, interval between last seizure and VEM, and seizure frequency. Statistical significance was defined as a p value less than 0.05. Microsoft Excel 2010 was used to generate histograms.

## Result

### Patients’ clinical and demographic characteristics

Of the 241 patients, 134 (55.6%) were male and 107 (44.4%) were female. The median age was 33.7 years old, with a range of 6–90 years old. The seizure frequency ranged from seizure-free to daily occurrences. Neuroimaging revealed responsible focal (IED-related lesions) in 23, nonspecific abnormalities such as demyelination of white matter and brain atrophy in 55, normal in 104, and unknown in 59. There were 191 patients with focal epilepsy and 50 with generalized epilepsy. Table 1 shows the demographic and clinical characteristics of these patients.

**Table 1:** Demographic and clinical characteristics of patients

	All patients	Focal epilepsy	Generalized epilepsy	*p*-value
N	241	191	50	
Female, n(%)	107(44.4%)	80(41.9%)	27(54.0%)	0.47
Age of seizure onset, years median(IQR)	28.0(15.9–51.0)	34.2(20.2–55.0)	13.2(10.3–20.4)	0.00^*^
Age, years median(IQR)	33.7(20.1–54.9)	38.9(25.4–58.8)	16.8(13.0–25.7)	0.00^*^
Seizure frequency, per month/ median(IQR)	1.0(0.2–3.5)	1.0(0.2–2.6)	1.0(0.3–20.0)	0.27
Interval between the last seizure and VEM, days/ median(IQR)	9.0(4.0–20.8)	9.0(4.0–21.0)	9.0(4.0–21.0)	0.33
Latency to first IED, minute median(IQR)	49.0(17.0–215.5)	63.0(19.0–299.0)	30.0(12.5–62.0)	0.00^*^

^*^ Significant *P* value

*IQR* Interquartile range, *VEM* Video electroencephalographic monitoring, *IED* Interictal epileptiform discharges

IEDs were detected in 222 of 241 patients (92.1%), with a median latency to the first IED of 49.0 minutes. The latency data was not normally distributed, with an early skew and a long tail of later discharges. IEDs were found in 53.5% (129/241) of patients within the first hour, 75.1% (181/241) within the first 4 h, 80.9% (195/241) within the first 8 h, and 84.6% (204/241) within the first 12 h. In all patients, the first IED were captured in 56.4% (136/241) patients during wakefulness and 35.7% (86/241) patients during sleep.

**Fig. 1:**
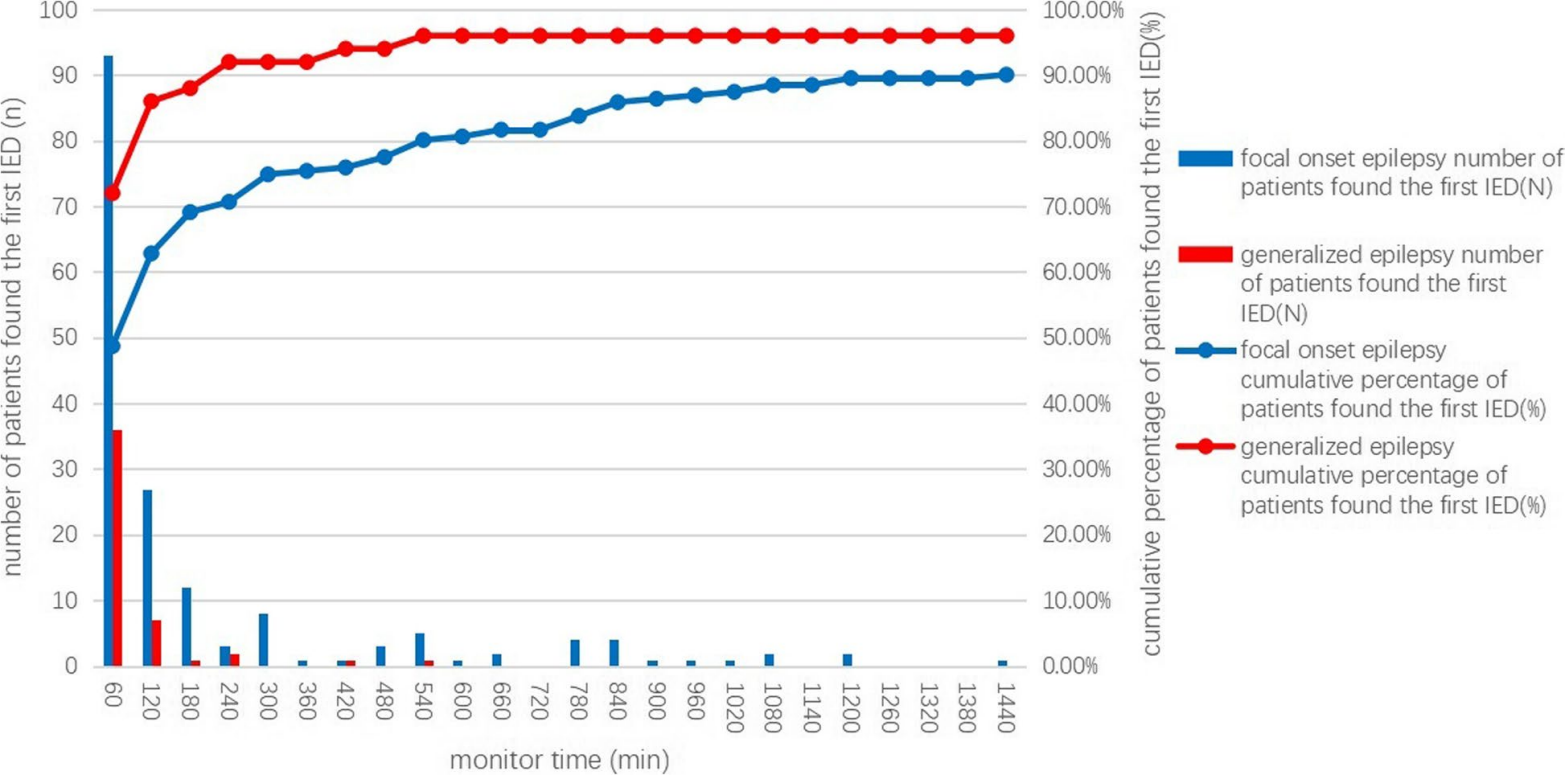
Time to the first interictal epileptiform discharge in patients with generalized and focal onset epilepsy

The median latency was 63.0 min in 191 patients with focal-onset epilepsy and 30 min in 50 generalized epileptic patients. There was a significant difference in the latency of the first IED between patients with generalized epilepsy and patients with focal epilepsy (*p* = 0.00). During the 24-h EEG, 93/191 (48.7%) of the patients with focal onset epilepsy captured the first IED within the first hour, 120/191 (62.8%) within 2 h, 148/191 (77.5%) within 8 h, 156/191 (81.7%) within 12 h, and 174/191 (91.1%) till the last hour. In generalized epileptic patients, 36/50 (72.0%) had the first IED found in the first hour, 43/50 (86.0%) within 2 h, 48/50 (96.0%) within 9 h, and so on until the end of the monitoring period. (Fig. 1).

Temporal lobe epilepsy is the most common type of focal epilepsy. Out of the 191 patients who had focal epilepsy, 146 had temporal lobe epilepsy, 20 had frontal lobe epilepsy, 3 had parietal lobe epilepsy, 8 had occipital lobe epilepsy, and 14 had multifocal epilepsy. There was no significant difference in the latencies of the first IED between patients with temporal lobe epilepsy and patients with frontal lobe epilepsy (median 67.0 vs. 53.5 min, *P* = 0.53). Similarly, there was no significant difference in first IED latencies between patients with temporal lobe epilepsy and those with other types of focal epilepsy (median 67.0 vs. 58.0 min, *P* = 0.29).

Using K-M survival analysis and the log-rank test of single factor analysis (Table 2), we selected factors with p ≤ 0.2 to enter COX multivariate analysis (Table 3). The COX multivariate analysis included gender, age of seizure onset, seizure frequency, interval between last seizure and VEM and type of epilepsy. Finally, the age of seizure onset, interval between last seizure and VEM and type of epilepsy were all found to be independently related to the latency of the first IED.

**Table 2:** Result of Kaplan–Meier survival analysis

Factor	Number	Median latency of the first IED (IQR)	χ2	*p*
GENDER			2.59	0.11
Male	134	43.5(17.0–280.0)		
Female	107	57.0(17.0–162.0)		
Age of seizure onset (years)			54.06	0.00
≤ 20	82	29.0(13.0–60.3)		
21–30	47	33.0(14.0–170.0)		
31–40	27	93.0(18.0–534.0)		
41–50	23	482.0(69.0–1440.0)		
51–60	31	161.0(32.0–608.0)		
> 60	31	50.0(21.0–122.0)		
Seizure frequency (/year)			6.38	0.27
≥ 365	27	77.5(23.8–227.8)		
12–365	60	47.0(19.0–94.0)		
2–12	98	63.0(17.0–271.0)		
1 -2	23	161.0(17.0–1440.0)		
0.5 -1	23	103.0(22.5–976.5)		
< 0.5	10	743.5(15.3–1440.0)		
Interval between the last seizure and VEM, (days)			22.79	0.00
≤ 7	98	34.0(13.0–122.0)		
7–14	59	54.0(19.8–142.0)		
15–30	38	67.5(17.0–466.3)		
31–182	35	65.0(19.0–475.0)		
183–365	3	1187.0(17.0,-)		
> 366	7	1440.0(1088.3,1440.0)		
Result of neuroimaging			1.50	0.68
Unknown	59	47.0(18.0–99.0)		
Normal	104	45.0(13.3–243.5)		
Responsible focal	23	30.0(18.0–163.0)		
Nonspecific abnormalities	55	73.0(18.0–534.0)		
Type of epilepsy			12.44	0.00
Focal epilepsy	191	63.0(19.0–299.0)		
Generalized epilepsy	50	30.0(12.5–62.0)

**Table 3:** Result of COX multivariate analysis

Factor	B	SE	Wald	P	Exp(B)	95% CI for Exp(B)	
						Lower	Upper
Gender	.122	.222	0.304	.58	1.130	0.732	1.745
Age of seizure onset	-.012	.006	3.861	.049^*^	0.998	0.977	1.000
Seizure frequency	-.002	.003	0.680	.41	0.998	0.992	1.003
Interval between the last seizure and VEM	-.002	.001	6.129	.01^*^	0.998	0.997	1.000
Type of epilepsy	1.072	.365	8.619	.00^*^	2.920	1.428	5.971

^*^ Significant *P* value

The first IED was discovered within 12 h in 204 of the 241 epileptic patients studied, whereas no epileptiform discharges were detected in another 37 patients. We analyzed the demographic and clinical characteristics of these two groups of patients and observed significant differences in seizure frequency, interval between the last seizure and VEM (Table 4). This is insufficient for predictive modeling at this moment due to the limited sample size. It would be beneficial for epilepsy diagnosis, if predictive models could be created to predict the chance of detecting IEDs in certain patients in later studies.

**Table 4:** Demographic and clinical characteristics of patients with and without IED within 12 h

		latency to the first IED ≤ 12 h	latency to the first IED > 12 h	*p*-value
N		204	37	
Female, n(%)		95 (46.6%)	12(32.4%)	0.06
Age of seizure onset, years median(IQR)		24.2(15.0–49.3)	29.9(17.7–44.0)	0.50
Age, years median(IQR)		29.2(18.9–54.0)	35.5(25.5–51.6)	0.15
Seizure frequency, per month/ median(IQR)		1.0(0.2–4.0)	0.4(0.1–2.0)	0.04^*^
Interval between the last seizure and VEM, days/ median(IQR)		8.0(4.0–18.0)	12.0(5.0–176.0)	0.05^*^
Neuroimaging results, n(%)	Normal	90(44.1%)	14(37.8%)	0.24
	Nonspecific abnormalities	43 (21.1%)	12 (32.4%)	
	Focal lesions	21 (10.3%)	2 (5.4%)	
	Absence	50 (24.5%)	9 (24.3%)	

^*^ Significant *P* value

*IQR* Interquartile range, *VEM* Video electroencephalographic monitoring, *IED* Interictal epileptiform discharges

## Discussion

In clinical practice, the time it takes from the start of the examination to the capture of epileptiform discharges in electroencephalography differs significantly between individuals. Some patients may detect epileptic discharges immediately after starting EEG monitoring, but others require many hours of continuous monitoring before they may detect epileptic discharges. Prior to beginning EEG, we can estimate patients' initial discharge latency based on major clinical characteristics, according to our findings.

Our study revealed a significant difference in the latencies of the first IED between generalized epilepsy and focal-onset epilepsy. The latency to the first IED was significantly shorter in patients with generalized epilepsy than in patients with focal epilepsy. This is consistent with the findings of previous research [5–8]. Generalized epilepsy is more common in children than focal epilepsy. We also discovered that the age of seizure onset is significantly related to IED latency. It agrees with the finding that generalized epilepsy has a shorter latency to the first IED than focal epilepsy. The explanation of the difference is unknown, however it could be related to the distinct brain network mechanisms involved in the creation and transmission of epileptic discharges in different kinds of epilepsy. A prior, generally acknowledged work has demonstrated that a recordable IED in scalp EEG requires a minimum of 10cm [2] of synchronized cortical activity [9]. If the activated cortex is not large enough, it will not be sufficient to be recorded by scalp electrodes. Some focal epilepsies arise in deep structures such as the medial temporal lobe, where abnormal discharges are difficult to detect using scalp electrodes [10, 11].

We also found that the shorter the interval between the last seizure and VEM, the shorter the latency to the first IED. If the patient has a recent seizure history, we may be able to collect his IEDs quickly after beginning EEG monitoring. If not, he may need further monitoring time to collect reliable diagnosis evidence. Previous research has also shown a higher positive rate of an emergency EEG shortly after the onset of an epileptic seizure [12, 13]. A recent seizure may be a sign of uncontrolled epilepsy and active cortical electrical activity. However, we did not find a relationship between seizure frequency and latency to the first IED. The frequency of epileptiform discharges and seizure frequency may not be linearly correlated [14]. Some types of epilepsy, such as self-limited epilepsy with centrotemporal spikes (SeLECTS), have frequent IEDs but relatively infrequent seizures.

Temporal lobe epilepsy is the most common type of epilepsy in adults. Few studies have been conducted to determine whether there is a difference in the latency of the first IED in different types of focal epilepsy, and the results have been inconsistent [6, 15]. In our study, patients with temporal lobe epilepsy had a slightly longer IED latency than patients with extratemporal lobe epilepsy, even though the difference did not achieve statistical significance. We speculate that there may be two reasons behind this. First, the majority of people with temporal lobe epilepsy had mesial temporal lobe epilepsy, in which the epileptic foci were deeply located and difficult to identify using scalp electrodes. Second, because most of the patients in our study had EEG monitoring beginning in the morning, they required a long period of monitoring before they could sleep. A previous study on the circadian cycles of epileptiform discharges found that Spikes were more frequent in sleep than wakefulness. Patients with temporal lobe epilepsy had a wake- to- sleep spike rate increase compared to patients with extra-temporal epilepsy [16]. Could it be that individuals with temporal lobe epilepsy would benefit more from starting monitoring at night in order to catch epileptiform discharges earlier? Future research is expected to create the groundwork for choosing a more ideal screening timing for various epilepsy patients by expanding the sample size and adjusting the monitoring start time.

Sleep promotes seizures and epileptic discharges. Both the frequency and range of epileptic discharges increase in slow-wave sleep compared with wakefulness, regardless of whether the patient has generalized or focal epilepsy [17–20]. However, we found that most patients' first epileptic discharge was recorded during wakefulness. This may be due to the fact that most of our patients start monitoring in the morning without sleep deprivation. Most patients stay awake for a long time after the start of monitoring and fall asleep relatively late. We excluded patients who had taken ASMs in the three months prior to the EEG examination. Therefore, our study does not address the effects of ASMs on the EEG.

The medial latency to the first IED is 37.5 min, and 84.6% of patients detect it within 12 h of EEG monitoring. However, in practice, the first IED is insufficient; we need several subsequent IEDs with the same properties to confirm our conclusion. As a result, the monitor time should be extended in practice.

### Limitation

The limitation of our study is that EEG monitoring only lasts 24 h. This may not be enough for some types of focal-onset epilepsy. We can detect IEDs in more patients if we increase the monitoring time, especially in patients with focal-onset epilepsy.

## Conclusion

IEDs were discovered during 24-h EEG monitoring in 222/241(92.1%) of the epilepsy patients that were included. Generalized epilepsy had a significantly shorter latency to IED than focal epilepsy. Longer monitoring periods may be required in patients with suspected focal epilepsy, late-onset epilepsy or no recent seizures.

## Data availability statement

The data used is available from the corresponding author upon reasonable request.

## Abbreviations

IEDs: Interictal epileptiform discharges
ASMs: Anti-seizure medications
EEG: Electroencephalography
VEM: Video electroencephalographic monitoring
